# In search of an empowering and motivating personal wellbeing pathway for Finnish heart patients

**DOI:** 10.1186/2193-1801-3-475

**Published:** 2014-08-27

**Authors:** Hanna Tuohimaa

**Affiliations:** Laurea University of Applied Sciences, Nummentie 6, 08100 Lohja, Finland

**Keywords:** Empowerment, Motivation, Citizen centric care, Seamlessness, Support

## Abstract

In this article, motivation for healthy living is discussed, with a special focus on empowerment and responsibility. The individual and his or her needs and preferences are taken as the starting point with an aim to sketch a holistic picture of the elements needed in a health promotive care process from the perspective of the individual, to enhance empowerment and motivation to lead a healthy life. Health and wellbeing related services are presented as an exemplar of a structural element by which society can work as an enabler of wellbeing, taking co-responsibility of the wellbeing of the individual. It is argued that this requires that the services of public, private and third sector service providers from different fields are seamlessly connected and easy to access, with support and information available throughout the personally tailored care pathway. Also the knowhow and attitudes of all the actors must be thought of. These aspects of a good care process have been identified through interviews with heart patients and workshops with actors in a region in southern Finland, as well as based on theoretical background information and prior research. These aspects form the personal wellbeing pathway model that is presented in this article as an ideal type of a service pathway to enhance taking actively charge of one’s own wellbeing.

## Introduction

Making choices has become a central element of the everyday (see eg. Beck and Beck-Gernsheim 2002). From the point of view of the society the choices we make may be unwelcome; we may choose to smoke, drink and eat unhealthy foods and neglect our health. Therefore we need ways to motivate healthy lifestyles and enable healthy choices in everyday life: exercising, following a low fat diet and refraining from tobacco and alcohol.

Even with behavior related illnesses being healthy is not solely a question of choice, however. There are factors such as genes that are completely beyond our control and factors such as childhood living conditions and poverty that shape our future and limit the choices available. Factors such as taxes on tobacco and the availability of restaurants serving healthy foods influence the choices we make. Therefore health and wellbeing cannot be viewed solely as a question of personal choice but also structural factors must be acknowledged (Stokols 1992). This is the starting point for the settings approach in health promotion, for example (Dooris 2005).

In this article, the health and wellbeing service sector and its agents are seen as one (but not the only) structural element in society that can have a positive effect on the wellbeing of the individual, and the choices he/she makes (in line with e.g. the goals of the Health Promoting Hospitals approach, Groene 2005). The concrete way this can be done is through the services offered and through organizational changes supporting the service production. The vision is an empowering care process that motivates healthy living, having a proactive effect on the wellbeing of the individual without coercion, focusing on the assets of the individual instead of the flaws and offering support when it is needed.

In accordance with Antonovsky (1996) health and wellbeing are seen as a continuum where the possibility of improvement and deterioration is always present. Therefore, health services and care processes are examined from a health promotive perspective throughout the care process, irrespective of the present health status. Following Banyard (1996) health is considered as a biopsychosocial system where besides physical aspects, social and mental aspects also need to be considered to understand the individual as a holistic entity and not just the carrier of a disease or specific symptoms. Therefore overcoming sectoral boundaries and tailoring the care process to meet the needs and desires of the individual are seen as necessities in promoting health and wellbeing.

In this article, a model for a motivating and empowering caring process is presented from the viewpoint of the citizen, with heart health as its frame of reference. The focus is on the needs and preferences of the individual and what the service providers can do to aid him/her in the pursuit of health related goals. Less attention is placed on the other side of the issue, i.e. to what the individual can do for him/herself. The purpose of the article is not to present a novel way of organizing health services. Instead, the aim is to point to elements needed in the ideal caring process from the user perspective, besides the actual medical treatment steps to promote health and wellbeing.

The origins of this paper lie on fieldwork among heart patients and service providers in a region in southern Finland in 2008–2010 as part of a project funded by the local university of applied sciences to improve the care of the heart patients in a proactive manner. The fieldwork was conducted by nursing students as part of their studies. One of the aims of the project was to provide the students with real life opportunities of learning (Raij 2013) at the same time responding to a need identified in the region to discuss what the health care sector and other actors in the region should do in collaboration to improve the care of the heart patients and anticipate future changes in the region proactively.

As part of the fieldwork, a survey was first conducted to the members of the local heart association (n = 206) about their experiences during their treatment. To get a deeper understanding of the experiences, further interviews were conducted to those respondents that were willing to participate (n = 64). For the actors in the region, four workshops were organized. During the fieldwork, it became apparent that health related services were not integrated from one service provider to another combining health promotion and prevention to the care pathway and the rehabilitation phase seamlessly. Instead, unmet service needs were identified. This information was used as the baseline information in the design of a new project funded by the European Regional Fund. In the project, the goal was to design a citizen centric framework for seamless care and pilot new wellbeing services to patch the gaps found in the path (Tuohimaa et al. 2012).

Combining empirical findings with theoretical knowledge on citizen centrism, empowerment and motivation, the ideas presented in this paper culminate in participation, information, support, seamlessness and attitudes and knowhow as the pivotal elements of the caring process besides the actual medical treatment. As a result, a holistic picture of the caring process emerges where all the resources of the different service providers can be better orchestrated to respond to the needs and preferences of the citizens in their health and wellbeing related endeavours. In this paper, the focus is on the citizen’s side of the path with only minimal reference to the actual organization of the services. The model points to the aspects in care that need to be considered to reach a citizen centric and empowering caring process. To transform the model into a concrete operations model for the regional organization of services still needs further development.

### Turning the focus to the citizen

With the prevalence of chronic diseases increasing there is a need to develop the health care system from treating acute health problems to supporting the self management skills of the patients (Funnel and Anderson 2004). This requires changes in the way health care is organized. For instance in the chronic care model (Wagner et al. 1996), system level requirements for better managing chronic diseases include explicit guidelines and care plans with a focus on self management, appropriate organization of care teams, education and decision support for the personnel and information systems and registries. The expanded chronic care model applies the model to the context of health promotion emphasizing the need for orchestrated action throughout the community to bring about health promotive effects (Barr et al. 2003).

The need for collaboration and integrated care is acknowledged in many countries (Leatt et al. 2000). In Finland, the national health archive is under development to smoothen the data flow from one service provider to another. Also new ways of organizing primary and special health services are sought. In the future, there will be five social welfare and health care regions in Finland that will be responsible for the organization of health and welfare services. At the moment, however, the Finnish health care system still consists of several subsystems with their own organizations and information systems. Primary health care is supplied by the local municipalities and special health care by larger hospital districts. All the Finnish residents are entitled to these public services with certain fees and day charges. The use of private health care is also partially subsidized and workers and entrepreneurs are entitled to occupational health care as part of the public health insurance. The local municipalities are responsible for arranging health services but the actual production of services can be outsourced to the private sector. Also non-profit organizations such as local associations offer services e.g. for peer support. Within one organization service encounters may follow the organization’s care pathway model smoothly and function well but when dealing with several service providers, as often is the case, orchestrated action is needed to guarantee seamless care.

With the change in perspective from organization centric acute medicine to a more holistic self-management oriented health care, the role of the individual is emphasized. As opposed to the early days of modern medicine, the idea of the patient as a person has become normative and resulted in the emphasis on autonomous choice and informed consent (Campbell 2013). Citizen centric health care manifests both at the individual level (Munthe et al. 2012) as well as in service development in general (Crawford et al. 2002). On the individual level citizen centrism is about decisions made in the actual care pathway of the patient. With methods such as shared decision making the service providers and the individual work together to find a solution to the issue at hand (Charles et al. 1997). On the service development level citizen centrism is about responding to actual user needs when designing services and service processes. Perfect fit of services to people with different backgrounds and life situations cannot be achieved without flexibility and agility designed into service processes.

The citizen perspective often presents itself in the form of highlighting personal choice. However, scholars such as Mol (2008) argue that there is altogether too much emphasis on personal choice. She claims that focusing on choice implies responsibility for the choices made. She argues that in the clinical practice a balance between wants and needs is sought in a continuous process of trial and error where mistakes and poor choices are accepted but surpassed as the focus is on the future possibilities, not past errors.

However, taking personal preferences into account need not imply responsibility for the choices made in the care process or in everyday life in general. Responsibility can also be interpreted as a forward looking concept of taking action instead of a backward looking concept of blame and accountability for the choices made (Cappelen and Norheim 2005; Waller 2005). The patient benefits from active engagement in the treatment process and taking charge of his or her own health. However, focusing on the responsibility as being guilty of one’s situation or the choices made is not fruitful. It may even have a negative effect on healing and on the doctor-patient relationship (Waller 2005). With a take-charge interpretation of responsibility, the focus is on the choices available at the present and the ways in which choices enhancing health and wellbeing can be motivated in the future.

Despite the citizen centric focus in health care, the role of the social determinants of health should not be downplayed. Health is not just about personal choice and individual preferences. The distribution of money and power, the daily living conditions and access to care among other things have a clear consequence to the health of the populations (CSDH 2008). However, as Devisch (2012) points out, views of personal or structural factors influencing health and wellbeing need not be seen in conflict. To be responsible in its etymologic sense means to encounter or be engaged in something coming from elsewhere which makes personal responsibility a contradictory statement in itself. In Devisch’s view personal and structural responsibility are two perspectives intertwined, mingled together forming the co-responsibility of both the individual and the service providers that escapes the binary logic of either-or.

Although the individual is the key actor in his/her own wellbeing and may be seen as responsible for taking action, the local community, the service providers and the society on the whole need to support and aid him/her when needed. This is not just a question of the health care sector but also other actors need to be involved. Especially when the goal is to have an influence on the social determinant of health concerted action of the whole society is required.

The approaches to health promotion such as the Health in All Policies approach and the Healthy Settings approach emphasize the role of the whole society in promoting health and enhancing wellbeing. The Health in All Policies approach focuses on influencing the policies outside healthcare to affect the determinants of health, both societal, structural factors as well as individual, lifestyle related factors. The focus is on the population level changes and especially on affecting health inequalities. (Sihto et al. 2006) For instance cigarettes and food that are major lifestyle factors behind cardiovascular diseases are globally manufactured and marketed products that need close international collaboration to be controlled (Jousilahti 2006).

The settings approach on the other hand focuses on the level of organizations and contexts of everyday life. It also sees health to be determined by a complex interplay of factors outside of healthcare: the environment, the organizations and personal factors, too. The different settings (schools, workplaces, hospitals, cities) are seen as dynamic, overlapping and complex systems where change is difficult to be measured as it is in the processes, not in the single interventions in themselves. To promote health in these settings changes are needed throughout the organization (Dooris 2005).

For example, the health promoting hospitals approach emphasizes the role of hospitals in the prevention of non-communicable or chronic diseases for the healthy population and in the improvement of self-care for the ones already affected. The aim is to make the patients the coproducers of their health and provide the patients as well as the citizens in general with adequate information and guidance and easy access to care. The hospital can also collaborate in the community as well as advocate for supportive legal regulation (Pelikan et al. 2013).

### Empowerment and motivation

Empowerment is seen as a key concept in promoting wellbeing in society. For example Gibson (1991) sees empowerment as a process of “recognizing, promoting and enhancing people’s abilities to meet their own needs, solve their own problems and mobilize the necessary resources in order to feel in control of their own lives”. A pivotal element of empowerment is the possibility to participate (Perkins 1995). In the health care sector empowerment may be seen as a relevant goal especially in the care of chronic diseases that need constant self-monitoring and making daily choices affecting health outside the doctor’s office (Funnel and Anderson 2004). The same daily self-monitoring is required in health promotion to make health related choices in the everyday.

Choice is embedded in the empowerment process, as decision making and choices in the everyday are seen as a prerequisite for taking responsibility of one’s own life. The ideology of patient choice and individual responsibility highlights the importance to match this power to decide with appropriate resources.

The feeling of control and having the resources and skills to make decisions form the basis of empowerment. It is often emphasized that individuals can only empower themselves, as empowerment is about taking charge of your own life. However, the living environment or the service environment can foster and support empowerment. Methods such as motivational interviewing have been developed to help people find solutions to their health problems themselves (see Rubak et al. 2005 for a review). To reach a citizen-led and flexible service structure participative methods are needed also in service development in general.

Some think that empowerment cannot be pursued within the health care sector but should be advocated by the lay community (Skelton 1994). From a service provider view point empowerment may be seen as exercising choice within the health system instead of the original meaning of independence from that system. On the other hand private sector consumers who value their provider/consumer relationship may want to concentrate on improving it instead of challenging it. As people’s needs and preferences vary, different approaches and viewpoints are needed (McLean 1995). Empowerment can also be used as an ideological rhetoric and as a means to pursue a multitude of goals in politics (Perkins 1995). However, contradicting uses of the term may also be seen as a positive thing in arousing public debate on social issues (Rappaport 1995).

Empowerment efforts may include interventions such as support groups, educational opportunities and changes in health care services. For instance health literacy skills i.e. skills to understand, evaluate and act upon health related information are seen as a critical to empowerment (Nutbeam 2000). Empowerment may take place directly through improvements in individual decision-making efficacy, disease complication management and improved health behaviours and indirectly through strengthened support groups, caregiver empowerment, enhanced satisfaction with provider/patient relationships and better access and efficient use of health services (Wallerstein 2006).

As health promotion is also about structures such as policies and practices, an individualistic approach to empowerment is not enough, also community empowerment is needed (Gibson 1991). On the community level empowerment is about joining forces to gain power in decision making and striving for a common good (Laverack 2006). In community development work empowerment may be pursued through user sensitive and participative service delivery; capacity building for raising knowledge, awareness and skills; advocacy for shared goals such as equality and the eradication of poverty; and social mobilization for placing demands, networking and participating in decision making (Schuftan 1996).

Measuring empowerment outcomes depends on the situation, as empowerment takes different forms for different people in different contexts. Zimmerman (1995) categorizes individual level empowerment into intrapersonal components, such as perceived control, competence and efficacy, interactional components that refer to understanding how the system works, such as problem solving and decision making skills and behavioral components that refer to actions taken to make a change. On the community level empowerment outcomes might include evidence of pluralism, new organizational coalitions and better access to community resources (Perkins & Zimmerman 1995).

Besides a goal in itself, empowerment may also be seen as a motivational tool for promoting healthier lifestyles. Feeling competent and being able to make autonomous decisions are themes that surface constantly in motivation theories. For instance, according to social cognitive theory self-efficacy beliefs (i.e. beliefs about the ability to exercise control over one’s actions) are crucial in attaining goals. People with higher self-efficacy beliefs set higher goals for themselves and expect more favorable outcomes. They are also more persistent when facing impediments (Bandura 2004). Several studies link self-efficacy beliefs to better motivation in health practices (see e.g. Marks and Allegrante 2005 or Strecher et al. 1986 for a review). Empowerment enhances the possibilities to take control of one’s life which in turn improves intrinsic motivation for change. Improving people’s self-efficacy beliefs and their feeling of control could then be seen as motivating changes in health related practices. Also the feeling of relatedness influences motivation (Vallerand 1997). It should be noted that empowerment may only be enabled, not forced upon the individual. The same goes with motivation, as intrinsic motivation is generally seen as preferable; extrinsic motivators need to be carefully applied (Cameron and Pierce 2002).

Motivation affects the direction and persistence of action and the effort put in goal achievement (Locke and Latham 2004). Antonovsky’s (1996) term “sense of coherence” is a generalized orientation toward the world as meaningful, comprehensive and manageable that facilitates movement towards health. Making sense of the world, understanding what is happening, having motivation and appropriate resources to pursue health related goals would then be essential in leading a healthy life even when confronted with a stressor.

It should be noted that empowerment in itself does not guarantee a healthier lifestyle. Also the perceived risk affects motivation. Without awareness of risk self-efficacy is irrelevant; there is no need to take action if no risk is perceived. On the other hand risk awareness combined with low perceived efficacy easily leads to denial, not action. Action is likely only when both the perceived risk and the perceived efficacy are high (Rimal 2001; Witte 1992).

In health care and health promotion in particular, the risk approach to health and trying to affect the risk awareness of the population is very common (Skolbekken 1995). Approaches that emphasize the role of individual health resources question the salience of focusing only on the risk factors; they see strengthening the positive at least as important as diminishing the negative (Hollnagel and Malterud 2000). Positive goals may work better than merely trying to diminish the risks. However, the motivational focus may also vary, both according to the situation as well as according to the person. Motivation may be directed towards approaching pleasure or accomplishment (promotion focus) or avoiding pain or failure (prevention focus). (see e.g. Higgins 1998 for a review of studies on the matter). This difference might be of importance e.g. in tailoring health messages either towards achieving health goals or avoiding illnesses or in offering either upward or downward comparisons or role models (Schokker et al. 2010). However, as Link & Phelan (1995) point out, even when talking about the risks of getting ill, we also need to look behind the risks and look at the social conditions that put a person at the risk of being at risk.

In the Personal Wellbeing Pathway model personal health goals are taken as the starting point in the care process. This gives the individual more control over her care. Other proponents of an empowering caring process vary according to the situation. A general outline of the elements needed based on the interviews and other materials is presented in the following chapters.

Although in the Personal Wellbeing Pathway Model the focus is on individual level empowerment, the model also acknowledges the role of community level empowerment in participating in service development and assuring that the services offered are just and valid for the health needs present in society and that alternative options for professionally led services are also available, within the limits set by law.

### Experiences on the care pathway of heart patients

With the vision of an empowered and motivated citizen who takes actively charge of his/her wellbeing the big question is how the health care sector can support and enhance this charge taking. Theoretical literature and prior research suggest participation and strengthening the resources of the individual as key elements in enhancing empowerment. Empowerment and motivation on the other hand seem to be linked through a shared idea of being in control. To find out what is needed of a good care process from the user perspective, we will now turn to the fieldwork that was conducted for the case group of heart patients.

During the fieldwork, a survey was first conducted to the members of a local heart association with 206 respondents (37% of the members) in the spring 2009. In the survey, the respondents were inquired whether they would be interested in participating in further interviews. During the fall 2009 66 of the respondents were then interviewed, of which 64 were heart patients themselves. These interviews of 64 heart patients form the primary data of this article.

The interviews were semistructured covering issues related to the different phases of the care process and how they were experienced, what kind of support and guidance would have been necessary in each phase and also about the changes in lifestyle after getting sick. The interviews were conducted by nursing students and therefore the interview form was detailed to guarantee consistent interviews with several student interviewers. The students made notes either on paper or directly to an electronic form. None of the interviews was recorded nor transcribed verbatim.

In the analysis phase, the open answers were categorized into subthemes within each question. The analysis was inductive and the themes derived from the data. After question specific analysis key themes were identified that responded to the research question: what are the elements of a good caring process from the point of view of the individual. In this article, the results are presented as the starting point of the model development work. The results are first outlined in this chapter on a more general level and in the following chapter in detail offering examples of the necessary aspects of a holistic caring process aiming at empowering and motivating ends.

Of the 64 interviewees 28 were women and 36 men. Most of the interviewees were between 61 and 80 years old. 28 of the interviewees had gone through a heart attack and 53 had gone through some procedure, e.g. by-pass surgery or coronary angioplasty. On the whole, the interviewees represented a content group of seniors who were quite happy with their treatment so far and of whom only seven considered that they had major problems with their health at the time of the interview.

The majority were content with the treatment they had received in emergency situations, in the hospital and at the clinic. 27 of those who had experience of the ambulance drive were content and 12 had had some problems e.g. with time delays and not being listened to. 58 of the interviewees had experiences of being in the hospital. Of those 40 were content and 9 had mixed feelings with being content with some parts of the treatment while not with others, e.g. when changing from one ward to another. 9 of the interviewees expressed solely discontent. They reported wrong diagnoses, time delays, lack of guidance and too busy personnel with too little time to engage with the patients. 47 of the interviewees had experiences of going to the outpatient clinic. 30 reported receiving good or sufficient care. Most discontent was due to long waiting times or having difficulties in receiving treatment (9 responses). There were also experiences of bad service and unpleasant behavior of the health care personnel (5 responses).

Generally the care pathway seemed to operate quite smoothly in the hospital, until the patient was discharged and had to start living again. Although there were many that had had good experiences in the rehabilitation phase, too, the most discontent was clearly present with relation to the rehabilitation phase. The interviewees had a variety of experiences of guidance and support in their treatment: guidance for nutrition (36 respondents), exercise (26) or healthy living in general (19) and discussion support (19). 21 reported regular checkups at the health center, 14 physical rehabilitation and 12 had also received peer support.

Approximately half of the interviewees had some ideas of what they would have wished more during the rehabilitation phase. They would have wanted more rehabilitation (9 respondents) with heart specific rehabilitation groups or rehabilitation phases. More information was required on what living with a heart disease meant (9 respondents), what to eat, how to exercise. Emotional support in a demanding phase in life was also needed (6 respondents). Some thought that they had not received any rehabilitation at all so they would have appreciated anything (4 respondents).

At the heart of this feeling of discontent with the rehabilitation phase lies the fact that in the Finnish health care system, in the hospital, care is administered by the hospital district where as in the rehabilitation phase care is administered by the municipality. This seam in the care process had clear consequences for the lived experience of being discharged and not knowing what to do next. One in four of the interviewees had had an occasion in their care pathway that they did not know who to contact or who was responsible for their care.

Another clear problem in the care process was the lack of emotional support. Some of the interviewees had had depression after their surgery, one described his feelings as if he had fallen into a void after discharge. As the treatment periods in the hospital are very short the patient may still be in shock at discharge when he is expected to take charge of his own treatment and make the contact with the local health center for checkups. Although more emotional support was required only in 6 open answers, in a closed question 37 of the interviewees considered that their treatment did not include enough emotional support.

Another issue related to the short treatment periods is the need for guidance and information on how to live with a heart condition. To lead a healthy life you need to know what you are allowed to do and also how you are doing at the moment. The period at the hospital being short precision is needed in the planning of the information provision.

Based on the empirical data, support, information and the seamlessness of care were considered as crucial for a good care process. As the responses show, however, also an otherwise good caring process may be considered as bad if the personnel are unfriendly or discount your knowledge. Therefore, a good caring process is also about attitudes and service experiences. Based on the analysis, these themes have been identified as the key elements of a good caring process that form the core of the Personal Wellbeing Model. The role of participation and engagement in the care process was added to the Model based on literature. The data was then read through again and the theme identified in the data deductively. Theme specific results of the data are presented in the following chapter.

Besides the survey and interviews for the members of the heart association, a set of workshops were also arranged for the health care providers and other interested parties to discuss future development of wellbeing services in the region. As the first step of responding to the problems in the heart patients’ rehabilitation phase apparent in the interviews, the position of a heart nurse was established in the health centers, to work as the contact person for the heart patients.

In the workshops, alternative future paths for the wellbeing of the region were sketched and their consequences discussed. In the end, a shared vision was formulated to proactively promote health in the region and implement a common operations model. The importance of cooperation between actors was acknowledged. The attitudes and knowhow of both the care personnel and political decision makers in the region were highlighted as a crucial step in forming a regional care pathway with a health promotive focus. When resources are scarce, acute problems requiring instant measures easily surpass health promotive ends. The same goes with offering rehabilitative services after the acute phase has been taken care of. At the same time health promotion and the need for personal involvement also require attitudinal change from the residents.

The workshops highlighted the need to take a proactive stance toward care and emphasize the health promotive phase of the care pathway. The Personal Wellbeing Pathway model presented in this article is derived from these empirical findings and offers the framework for constructing a citizen centric care pathway in the region.

### The personal pathway towards health and wellbeing

The Personal Wellbeing Pathway Model describes what is needed of a good care process from the point of view of the individual with the goal to empower and motivate healthy living. It should be noted that the focus is not on the actual medical treatment procedures per se but what is needed in addition. In the model, the individual is the most pivotal actor. The role of the service providers is to supply the individual with appropriate resources and support to take charge of his/her own health and wellbeing, the starting point being a self-set goal or a future vision and the outlining of an action plan for goal attainment. It is argued that in an environment of a multitude of service providers the services of different services providers must be seamlessly connected and easy to access, with support and information available throughout the personally tailored wellbeing path in order to motivate and enhance empowerment. Also the knowhow and attitudes of all the actors must be thought of.

Besides the individual and the service providers, the local community (family and friends) also has an important role in enhancing wellbeing. Also structural and societal factors influencing health and wellbeing on the general level need to be acknowledged. It is suggested that all the actors influencing a person’s care and wellbeing must be acknowledged to make use of all the resources in society that can have a positive effect on wellbeing. One does not have to be a health care professional to pursue wellbeing related issues; teachers, neighbors, volunteers, employers and other actors in the region may be engaged, too. At the same time it is pointed out that also the structural aspects such as living conditions and laws and regulations have an effect on individual wellbeing and form the basis for either healthy or unhealthy living.

The core of the pathway consists of the individual and his/her specific characteristics and the concrete treatment steps and activities relevant to the life situation, in health care consisting of health promotion, treatment and rehabilitation of one or several health conditions. The pathway includes the actions of the individual, the local community and the actions of the service providers and other actors relevant in the specific life situation, including both public and private sector for-profit and non-profit actors. It should also be noted that with an emphasis on health promotion the phases of treatment and rehabilitation may be all together avoided. Besides treatment steps, the aspects of participation, support, information, seamlessness and attitudes and knowhow must also be acknowledged in the wellbeing pathway. The importance, content and scale of these aspects vary according to the life situation and background of the person with whom the model is implemented.

**Figure 1:**
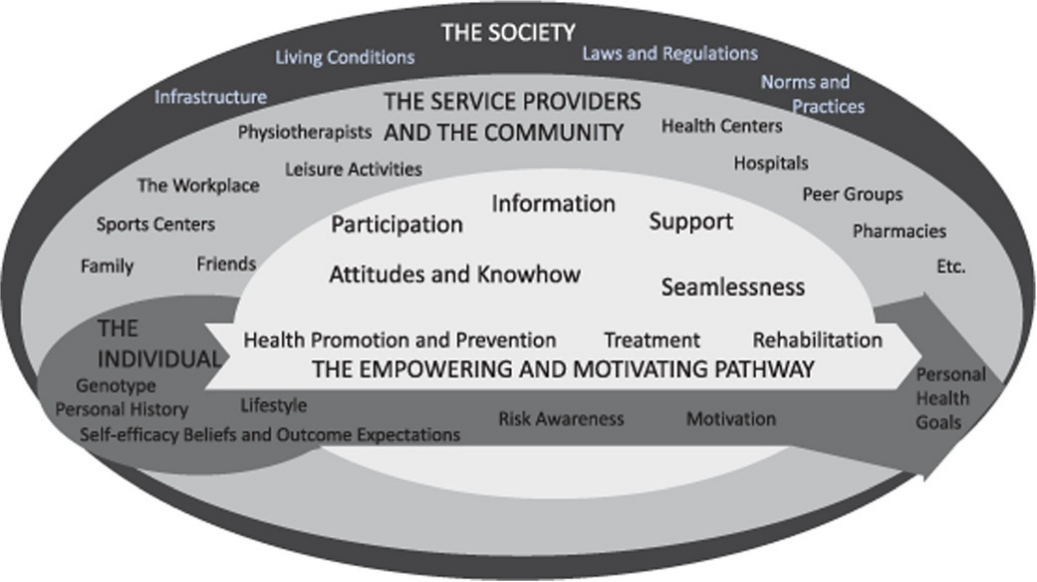
**The personal wellbeing pathway model.**

Although different path options may be defined beforehand the tailoring of services should always be done to suit the specific case. With a citizen centric perspective top down definitions should be flexible. The individual has the best knowledge of his/her motivators and aspirations although support may be needed in clarifying relevant health related goals and defining appropriate steps for their attainment. The professional’s role is to combine the aspirations with the real world options. Ethical questions may arise when the individual has preferences that do not fit the medical guidelines.In the following subsections, the five aspects of the wellbeing pathway are described with examples from the fieldwork on heart patients. It is not suggested that these aspects are or should be separate elements in the care process. Instead, they are intertwined and can be put into practice in many different ways (Figure 1).

#### Participation

A pivotal element of empowerment is the possibility to participate (Perkins 1995). In the interview data on heart patients, the interviewees were quite content with the care that they had received. The need to participate in defining the care plan was not discussed in itself. However, the dissatisfaction that existed with the rehabilitation phase was caused by a deficiency in the care process design; the dissatisfied interviewees generally wished for more support and guidance on what they were allowed to do. The rehabilitation phase did not respond to their needs and would have benefitted from tailoring either at the individual level or considering the amount of dissatisfaction that existed, also on the level of the care process on the whole. There were also some interviewees who claimed that they had not been listened to and hence had not received the treatment they would have needed in time. These patients would have wanted to participate in making their treatment plan but were dismissed.

In the data, the importance of participation was most evident in situations where more individual care process design would have been needed. As long as the care process operates well and the patient is satisfied with the treatment, the role of participation is not as evident to the patient as when problems occur. With opportunities for participating in tuning the care process available throughout the care process and support for recognizing one’s assets in decision making, a take charge attitude towards one’s health may be pursued.

Participation may be considered both at the level of service development and at the level of tailoring the available services and care processes to meet the needs of a specific individual. The Personal Wellbeing Pathway Model is primarily focused on the participation of the individual in the design of his/her own care process, although the need for participation in service development is also acknowledged. In the model, the starting point is a self-set goal or goals concerning health and wellbeing and a tailored set of services and actions to support goal attainment. The sense of direction one has in attaining the self-set goals depends on the perceived self-efficacy of the individual; the feeling of being able to control one’s own health and wellbeing. Outcome expectations take into account the perceived facilitators and impediments in the everyday (Bandura 2004). Tailoring the services to meet the needs of the individual must be done in close cooperation between the individual and the service providers. This can be done e.g. with the help of a care manager.

In the treatment process, the level of activity varies. For instance when the need for treatment is acute the individual has a less active role. It is also possible to mandate someone else to take control. In health promotion, rehabilitation and the management of chronic diseases, the individual has a pivotal role in making health related choices in the everyday. However, as the interviews indicated possibilities for participation need to be present in all phases of treatment, also in emergency situations. It is also crucial to find the threshold between enabling and demanding activity. Although the autonomy of the individual must be respected, support needs to be available if needed.

#### Support

Research links social support to lower cardiovascular mortality (Uchino 2004). Our fieldwork with heart patients indicated that heart patients needed most support on the rehabilitation phase. They felt in need of more guidance for everyday life with a heart disease and of someone to discuss their feelings and worries with.

Support strengthens the resources available to the individual. The individual needs support to set the health related goal, to find the right path to attain it and to keep on the path even when facing impediments. Support may include discussing treatment options as well as concrete, tangible help in the everyday life and self-management issues, and listening to the individual and offering emotional support and companionship (Uchino 2004).

Often the most support comes from family members. In the fieldwork, peer groups and other services local associations have were not appreciated as much as the doctors and nurses and their support although the patients in the study were members of the local heart association. The wish to get more support from the public health service sector collides with the lack of resources to meet the need. Therefore, other solutions such as the activities of the local associations have to be utilized more to respond to the need for support in the rehabilitation phase.

The individual needs support in all phases of the wellbeing pathway, both from the official professionals and peers and family members. The family members also need support in coping with the illness of a loved one. The society may also support the individual in many ways e.g. by offering rehabilitation services and other benefits, by constructing recreation facilities and bicycle lanes or by restrictions e.g. on the sale of tobacco.

The amount and type of support and guidance needed can be tailored to fit the life situation and the level of self-efficacy. If a person has a high sense of efficacy and positive outcome expectations, only minimal guidance is needed. People with self-doubt need more guidance and those with little feeling of control of lifestyle changes need the most structured programs to make healthy changes in life (Bandura 2004, see also Sweet et al. 2011). By empowering methods the level of self-efficacy and the sense of control can be improved. Obviously the severity of the health issues at hand and the overall life situation also influence the need for support and its content. In lifestyle changes different stages of change require different interventions (Ogden 2007, 21–22). Unrealistic expectations may lead to disappointment and a decrease in activity (Sweet et al. 2011).

Support with an empowering focus strengthens the feelings of control and self-efficacy which in turn enhance motivation for self-management and healthy living. On the other hand, no amount of support offered can make up for the absence of an intrinsic desire for change.

#### Information

In our fieldwork, there were several problems with information provision. The information systems of different service providers were often not interoperable and patient information was lacking. Many heart patients felt in need of more information during their rehabilitation phase; it is difficult to lead an active life if you don’t know what you are allowed to do. Knowledge of the consequences of different lifestyle choices is a prerequisite for a healthy lifestyle as without risk awareness there is no need for behavior modification (Witte 1992).

The patient associations participating in the workshops on the other hand had trouble reaching heart patients as they had no access to patient contact information. Instead, leaflets and notice boards were used to contact the patients at the health center and promote the peer support option to them.

Getting information about health habits and risks for health is essential for healthy living but only in so far as there are resources and beliefs in efficacy to take action and information about the care and self-management options. Follow up data is necessary to estimate the effects of treatment. Without information there is no need for action and no use of making goals as there is no way of knowing where one is headed.

The individual needs information on maladies and their treatment, on health and wellbeing, on his/her own condition and follow-up data to estimate goal attainment. The individual also needs information on the next treatment steps and the services available in the community. The amount and nature of information necessary depends on the situation and on the individual and therefore needs tailoring and sensitivity. As discussed earlier, the level of self-efficacy beliefs influences reactions to perceived risks. In some occasions too much information may lead to worry and anxiety (Malin and Teasdale 1991). An active information seeker may be happy with the information available on the internet whereas others prefer guidance from the health care professionals or peers. In this sense information may be seen as one form of support, as informational support (as defined e.g. by Uchino 2004).

Information provision is an easy way to affect self-management especially when there is a time constraint on the face-to-face encounter (Heisler et al. 2002). However, efforts to enhance the health literacy levels of the individual are needed (Nutbeam 2000) as well as efforts to make the information more understandable to the lay people.

From the health resource perspective (Hollnagel and Malterud 2000) information may also be seen from another viewpoint. As opposed to receiving information about the risks of specific lifestyle choices, information may also be seen in the context of getting information about the resources available at a specific moment from within, through reflection. This reflecting can be facilitated by the care personnel, as Hollnager and Malterud suggest, by specifically asking about what the individual normally does to stay or become well. In addition to offering retrospective knowledge to the client, it also offers new insight to the care personnel about the strengths of the client that can be utilized in the care process later on.

Besides the information that the care personnel receives from discussions with the client, the care personnel obviously needs information on previous conditions, laboratory results and other health history. When dealing with several service providers, it is essential that the flow of information from one actor to another is seamless with no time lag or information losses. Also methods of incorporating information provided by the individual (e.g. measurement data) to the official information systems are of importance.

#### Seamlessness

The interoperability of information systems is crucial for seamless care. One of the reasons why the heart patients in our fieldwork felt in need of more support on the rehabilitation phase was the fact that in the Finnish context the transition from hospital to rehabilitation also meant a transition from special health care administered by the hospital district to primary health care administered by the local municipality. This transition often lead to a gap in the information flow leaving it up to the patient to contact the local health center for checkups.

Seamlessness of services requires the cooperation of all the actors offering treatment, support or information, both official and unofficial actors and the clarification of roles and treatment processes. Both the individual and the service providers should always know what the next steps of the pathway are and who is responsible for the treatment at any given time. This requires the definition of possible service paths beforehand and acknowledging all the actors and their roles in the care process.

The need to incorporate health promotion more fully to the care pathway was a concern of the health care professionals participating in the workshops arranged as part of the fieldwork. Nine of the heart patients interviewed on the other hand thought that their condition was too acute and surprising to be prepared for beforehand. 36 of the interviewees did not feel in need of any guidance or information prior to their heart diagnosis. This is a basic question of prevention; how to prepare for the unexpected, how to motivate for lifestyle changes before anything happens?

With health care focusing more on prevention and health promotion new connections between health care, sports, community development, culture and other areas of life will be made. Health related issues may be pursued in a variety of settings outside the health center. The fact that public resources are scarce and service needs high as the population ages encourages cooperation between public, private and third sector service providers to respond to the health related needs of the individuals. Proceeding on the wellbeing pathway should be as easy as possible and moving from one service provider to another should be seamless without service discontinuities or information delays. The fact that care and service options are plenty and changes may occur during the care process brings about a challenge to make the chosen pathway seamless. However, a seamless and well organized service path makes achieving health goals easy which may improve motivation to pursue the goals further. Unclear procedures and unmet service needs may be seen as barriers to action that impair motivation. The less motivated the individual is and the less efficacy beliefs the individual has the easier it is to drop out of the service path when facing problems.

#### Attitudes and knowhow

In our fieldwork, the heart patients were generally quite happy with the care that they had received for their condition. However, some felt that they would have needed more emotional support for themselves or for their family members. Some suspected that the health care professionals were too busy to listen to their patients’ worries. If health and wellbeing are seen as a biopsychosocial continuum, the need for emotional support has to be acknowledged in the health care sector. As mentioned, cooperation with local patient associations may be seen as one possible solution to offering more support for the patients. Acknowledging the benefits of cooperation with local associations would lead to more interest in developing new structures for cooperation, making it easier for the associations to reach the patients in need of support.

Operating in an environment of a multitude of service providers in a health promotive manner that fosters the empowerment and motivation of the citizen requires certain knowhow, skills and attitudes from all parties of the wellbeing pathway. Service providers need constant training for new methods and theories that also require attitudinal change. This is crucial for the whole organization, including management (Kemppainen et al. 2012). There needs to be a shared understanding about what citizen centrism, empowerment and health promotion require from the organization. Guidelines such as the standards for health promotion in hospitals (WHO 2004) and the attributes for a health literate organization (Brach et al. 2012) help implement the required changes. With a multitude of service providers, there needs to be awareness of the necessity of cooperation and its benefits for all the actors as well as for the individual service user, too.

In the service encounter supporting empowerment requires e.g. mutual trust, respect and commitment (Rodwell 1996). For example Paterson’s (2001) study on diabetes patients indicated several practices that contradicted the stated goal of empowerment and active participation. The diabetics interviewed claimed that the practitioners discounted their experiential knowledge. They often felt that they did not have adequate resources to make informed decisions. The use of medical jargon, lack of time to ask questions, the dismissal of monetary constraints all hindered participatory decision making.

There are also demands for the individual, his/her knowhow and attitudes. With a health promotion focus in health services, the individual is expected to take charge of his/her own wellbeing, albeit with support from the health care professionals. Improving self-management skills and self-efficacy beliefs should be a top priority in all health and social services and in society on the whole to support individuals in achieving their goals and leading a healthy life. In order to manage all the information available the individual also needs health literacy skills in order to evaluate the information and transform it into gaining a greater control of one’s life (Nutbeam 2000).

On the society level there needs to be acknowledgement of the importance of participation in decision making and the relevance of the living environment in shaping healthy habits. An attitude of take-charge responsibility of the individual, an empowering attitude of the service providers and an enabling attitude of the society on the whole is needed to reach this kind of an operation’s model.

### Applying the model in practice

As the Personal wellbeing pathway model is citizen centric by definition, it is not bound to a specific health service organization structure. The model describes what kinds of elements are needed in the care process from the perspective of the individual, based on interview data of heart patients as well as literature. The model suggests that the elements should be provided for by the multitude of service providers and actors present in the municipality/region where the individual lives. The model leaves open the question of how care should be organized from the perspective of a specific service provider organization. Models such as the chronic care model (Wagner et al. 1996) that have been developed to attune service provider organizations from acute medicine to chronic care, might support its implementation.

Rather than organization specific the model describes a regional network of service providers and actors that are present in the everyday settings of the individual. In accordance with the settings approach of health promotion and the Health in All Policies approach the model acknowledges the role of the whole of society in supporting wellbeing and enhancing health.

The model is not bound to specific, individual services either. If the resources of the health service provider are ample, it is possible to provide all the elements described in the previous chapter through interactions with the patient and the health care professionals (doctors and nurses). This requires that issues such as the need for emotional support are acknowledged and the provision of information carefully planned, as well as the roles of the professionals and the patient considered. However, to provide support for the everyday, the resources of the health care sector would quickly dry out. The more responsibility there is on the individual to make healthy choices and improve one’s wellbeing the more important it is to offer support to the individual. The family has a central role in offering support but not all of us have family members to turn to. As family members need support themselves, too, they are not a limitless resource. Patient associations as well as all the everyday settings that people are engaged in are a worthy alternative for emotional support as well as other forms of support, coming both from peers as well as professionals.

Although the model is primarily concerned with human action (services or other activities) it should be pointed out that our everyday settings may be health promotive as places, too. The outer circle in the model describes this aspect of the society, as well as the policy aspect of healthy living. Although the service providers in the inner circle are mostly described as service providers, it should be noted that they can also have a role in the outer circle, as policy advocates for a healthier city, for example.

In the region of its origin in southern Finland, the model has been used as the framework of a project of the University of Applied Sciences developing health promotive and inclusive services in a citizen centric manner with a multiactor perspective during 2011 to 2014 as part of a larger cross regional European Regional Fund project. During the project, 19 members of the care personnel of a public health care provider in the region were interviewed using the elements of the model as interview themes (Tuohimaa and Ranta 2014). As a result, theme-specific recommendations for further development were made. These included taking personal health goals as the starting point of the care process, systematizing the collaboration with the third sector and the private wellbeing service providers, collecting data on available services to one location, finding ways to address contradictions between lay knowledge and official recommendations and founding new partnerships for providing everyday support for health related lifestyle changes. On the level of attitudes and knowhow, the interviews pointed to the need to emphasize guidance knowhow in addition to know how about the subject matter, to acknowledge the role of health promotion in all stages of life, including old age, to take co-responsibility of the continuity of care and to see collaboration as a resource instead of a time consuming burden.

Also measures to respond to these recommendations have been taken in the project. For example, an electronic service basket has been developed to integrate the services of the public, private and the third sector service providers into one virtual platform to be used with the patients of a case manager. A workshop round was organized to improve the seamlessness of care for discharged heart patients. Also information snapshot events have been organized to the care personnel based on theses with literature reviews of different guidance methods (e.g. group counseling, blood pressure coaching, motivational guidance). And finally, a multitude of different activities and service pilots have been conducted to bring health promotion to different settings, the kindergarten, school and care homes, for example (Tuohimaa and Pirilä 2014).

In the project, the Personal Pathway Model has been used both as the visionary ideal state of the pathway of the individual in the region that has been tuned for specific life situations and conditions as well as a concrete tool for checking that the measures taken in the project make note of all the elements of the model.

## Conclusion

With the citizen taken to the fore organization centrism may be avoided and a better fit of services attained in a region. By understanding user needs the care processes of different organizations may be attuned to gain a seamless fit. Although the citizens may and should be engaged in the design of the services in the end the responsibility for offering appropriate services in Finland is in the hands of the public sector with private services as the supplementary alternative. Through the Health in All Policies strategies the role of the government is highlighted in orchestrating all the organizations in society for the pursuit of the health and wellbeing of the nation and its citizens.

The different aspects of an empowering and motivating caring process form the checklist of what kind of things need to be considered besides the actual medical treatment when forming the regional care pathway or designing an individual care pathway from the user perspective. Analysing the present state of the services on offer and the user experiences reveal where the weaknesses of the service offerings in a specific region are and where focus should be placed.

Participative methods such as workshop rounds and discussion forums in applying the model enhance acceptance and attitudinal change needed in the change process. Besides health care professionals, other actors as well as citizens and potential service users also need to be a part of the model application and service design. Sensitivity to the present and possible future needs and preferences of the service users is essential for flexible and agile service production.

As a result of a citizen centric approach to health and wellbeing where the above mentioned elements of participation, support, information, seamlessness and attitudes and knowhow are acknowledged in the care process, empowerment in daily life and motivation to make healthy choices may be easier to achieve. Empowered citizens have a positive self-esteem, they are more able to set and reach goals, they have a sense of control over life and change processes and a sense of hope for the future (Rodwell, 1996).

With the focus of the article being on what the service providers can do to aid the individual in leading a healthier life, only minimal attention has been placed on what the individual can do him/herself. Constant monitoring of one’s health status is one of the easy ways of taking actively charge of one’s health. For instance, in the interviews, all but 6 of the interviewees new their blood pressure and cholesterol levels.

In the interviews, the heart patients were asked about the changes that they had made to their lifestyle after being diagnosed or suffering from a heart episode. About one in four had not made any changes, mostly due to already leading a healthy lifestyle, but also for reasons such as a desire to live life to the full. The ones who had made changes reported changing their eating habits, quitting smoking and exercising more. However, also the opposite happened, nearly half of the ones making changes to their lifestyle had had to cut back on their regular activities. Whether the changes were caused by the actual medical condition or just a precaution due to the evident uncertainty some had about what they were allowed or not allowed to do, cannot be estimated from the interviews. For some, the need to slow down was seen as an opportunity to enjoy the silence, in a sense improving their wellbeing as opposed to a previous stressful lifestyle.

With the enormous shock that a life threatening seizure such as a heart attack can cause for the individual, it seems obvious that most of us would be ready for substantial lifestyle modifications to prevent further episodes. To bring about a similar interest in lifestyle changes prior to any health problems is the key question of health promotion. Focusing on the resources and assets of the individual instead of risks and flaws brings about the possibility to find positive motivators for healthy living; many joggers appreciate the endorphins related to the jogging session in itself and need no other motivators.

However, it also needs to be acknowledged that individuals may have other priorities in their life instead of following the healthy lifestyle and the nutrition and exercise recommendations. An empowered citizen may be happy with his unhealthy habits and consider them having positive effects on his wellbeing; offering ways to relax, socialize or enjoy life. If health is considered to consist of mental and social aspects, too, we cannot focus solely on lifestyle choices related to physical health. Focusing too much on the healthy lifestyle may bring about anxiety and worry (Rangel et al. 2011). A balance in physical, mental and social wellbeing is needed.

The vision of a community that makes leading a healthy life easy and fulfilling is a goal that requires many changes in society that are beyond the control of one single organization or even the service sector on the whole. That is why approaches like the Health in All Policies are necessary. It seeks changes in different levels of society, international, national and local (Sihto et al. 2006). A shared vision is needed regionally as well as nationally and internationally in order to proceed and develop structures for collaboration. As most of our life is spent outside the doctor’s office, the way in which different every day settings can be turned into healthy living environments is crucial, as is acknowledging the role of the social determinants of health. Yet the health care sector can be one part of the solution, if the services available truly support the individual on matters close to heart, respecting and taking use of the assets and resources available for producing and maintaining wellbeing.
